# Methemoglobinemia Induced By Ingesting Lava Lamp Contents

**DOI:** 10.5811/cpcem.2018.5.38261

**Published:** 2018-07-16

**Authors:** Mary E. Funke, Chanel E. Fischetti, Anne M. Rodino, Stephen P. Shaheen

**Affiliations:** * Duke University, Department of Emergency Medicine, Durham, North Carolina; † UCI Medical Center, Department of Emergency Medicine, Orange, California; ‡ Duke University, Department of Medicine, Durham, North Carolina

## Abstract

A patient presented after ingesting the contents of a lava lamp that he believed to contain alcohol. It was later discovered that this product was comprised of 76% calcium nitrate, leading to his subsequent development of methemoglobinemia. This disease is a medical emergency secondary to poor transportation of oxygen and resultant tissue hypoxic effects. Therefore, having high suspicion for this disease process in patients with toxic ingestions, understanding the proper diagnosis, and promptly starting treatment are all critical actions for emergency physicians.

## INTRODUCTION

Methemoglobinemia can be genetic or acquired following exposure to various toxins. It can be induced by substances containing nitrates. When nitrates are metabolized, they form inorganic nitrites that act as the oxidizing agent on iron in hemoglobin. Methemoglobinemia occurs when iron in hemoglobin is oxidized from the ferrous state (Fe^2+^) to the ferric state (Fe^3+^). This prevents hemoglobin from binding oxygen, causing varying degrees of hypoxia.^1^

Acquired methemoglobinemia can be induced by ingestion of toxic substances such as paint thinner, which contain nitrobenzene, or it can be secondary to other less-common manifestations.^4^ For instance, in 1992 at a school in New Jersey, more than 40 children contracted methemoglobinemia after they ingested soup later found to contain high quantities of nitrite.^2^ There have also been reports of methemoglobinemia in septic patients thought to be secondary to large amounts of nitric oxide.^3^ In addition, medications such as sodium nitrite used to treat cyanide poisoning have been show to induce methemoglobinemia with toxic doses as low as 300mg.^4^

## CASE REPORT

A 71-year-old, 86 kilogram male with a history of alcohol abuse, dementia, chronic kidney disease, and hypertension presented to the emergency department (ED) after the ingestion of approximately half of a retail lava lamp’s contents. On-scene vitals by emergency medical services (EMS) were notable for 90% oxygen saturation on room air. The patient was placed on two liters of oxygen by nasal cannula (NC), and the North Carolina Poison Control Center was called; they recommended supportive care, laboratory studies, and an electrocardiogram (ECG) with continuous cardiac monitoring. The risk of toxic ingestion was thought to be low because of the recent manufacture date, which theoretically minimized toxic contents previously found in similar products because of regulatory changes.

In the ED, EMS reported that the patient had consumed the lava lamp because he believed it to contain alcohol. The patient was unsure of the time of ingestion, though all history was limited by his chronic dementia. Initially, he remembered having nausea and vomiting at home, but was asymptomatic on evaluation. On physical exam, vital signs were notable for a blood pressure of 129/68mmHg, heart rate of 74 beats per minute (bpm), and oxygen saturation of 97% on two liters NC. Patient was tearful but in no distress. He had equal and reactive pupils, his heart rate was regular, breath sounds were clear, abdomen was soft, and he had a normal cranial nerve exam. Family in the room reported he was at his baseline mental status: delayed speech and baseline dementia. They seemed unconcerned about any new or significant mental status changes.

Routine laboratory results were normal except for the following: white blood cell count 14.4×10^9^/liter, hemoglobin 10.0 g/dL, potassium 6.3 mmol/L, carbon dioxide 14mmol/L, blood urea nitrogen 37 mg/dL, calcium 12.1 mg/dL, creatinine 2.3 mg/dL, and anion gap 23 mmol/L. Serum drug screen was negative for ethanol, acetaminophen, and salicylate. A 12-lead ECG showed normal sinus rate, with concern for peaked T-waves in the apical leads. A chest radiograph was read as persistent low lung volumes with bronchovascular crowding and bibasilar opacities, likely reflective of atelectasis.

The patient was treated for hyperkalemia with calcium gluconate, insulin, dextrose, and sodium bicarbonate; the Poison Control Center was updated on the findings and initial treatments. Within three hours, the potassium had corrected to 5.5 mg/dL and creatinine increased to 2.8 mg/dL. His mental status was unchanged. He continued to saturate in the low 90s, originally managed with two liters NC, but later requiring slight increases in his NC needs. Initially, it was thought that his lower saturations were attributed to a possible aspiration event, especially with reports of vomiting earlier in the morning.

About six hours into his ED visit, while pending admission, the nursing staff called providers to the room for an acute change in mental status with concomitant aspiration event. The patient’s oxygen saturation acutely dropped to 85% on NC, and high-flow NC was initiated. The patient’s saturation remained 85% on the Masimo SET™ pulse oximeter despite oxygen supplementation, and he was responsive to only painful stimulation. On auscultation, his lungs were clear bilaterally without wheezes or rales; his skin, especially distal, appeared gray and mottled. His blood pressure was 101/52 mmHg, and he became tachycardic to 101 bpm. A non-rebreather oxygen mask was applied at 15 liters and albuterol was administered with no change in respiratory status. Repeat radiograph was unchanged and it was thought this could be due to aspiration pneumonitis or acute respiratory distress syndrome. However, with the acute change, a venous blood gas was then also sent with the following notable results: pH of 7.22, methemoglobin of 45.6% and a lactate of 2.7 mmol/L.

Given this finding and the fact that his oxygen saturation remained the same on supplementation, the patient was diagnosed with methemoglobinemia. Subsequently, he was administered two doses of methylene blue (50 mg intravenously) 20 minutes apart. Over the next half-hour, his color and oxygen saturation improved, followed by a return of his mental status to baseline at initial presentation. A follow-up arterial blood gas at 45 minutes showed pH of 7.28 and methemoglobin of 9%. The patient was admitted to the intensive care unit.

The Poison Control Center was later updated. After research and testing, it was discovered that the components of the lava lamp ingested included 76% calcium nitrate, 23% water, and 1% potassium enol.

**CPC-EM Capsule**

What do we already know about this clinical entity?

Methemoglobinemia is a serious condition resulting in tissue hypoxia.

What makes this presentation of disease reportable?

There have been multiple case reports of ingestion of toxins leading to Methemoglobinemia, however, this is the first case report of a lava lamp being the inciting event.

What is the major learning point?

Keep Methemoglobinemia on your differential in any toxic ingestion, especially those who appear altered and have signs of hypoxia.

How might this improve emergency medicine practice?

Having this insight can help detect this disease earlier and allow patients to get proper treatment.

## DISCUSSION

This patient survived after the ingestion of lava lamp contents later found to contain 76% calcium nitrate, which induced methemoglobinemia. It was originally presumed that the patient’s change in respiratory status was secondary to aspiration, especially because he was only requiring two liters via NC for the majority of his ED visit. However, after his seemingly sudden decompensation, a venous blood gas ended up being a crucial test to help come to the correct diagnosis. We believe that the delay in his respiratory decompensation was likely secondary to delayed metabolism of calcium nitrates, producing levels significant enough to be clinically responsible for his methemoglobinemia symptoms.

The drug of choice in the treatment of severe methemoglobinemia is methylene blue.^5^ In the setting of drug- or toxin-induced methemoglobinemia, the actionable treatment level is generally considered to be 20% methemoglobin in symptomatic patients and 30% in asymptomatic patients.^5^,^6^,^8^ Normal methemoglobin levels are 1% – 3%.^5^ Methylene blue is available as a 1% solution (10 mg/ml).^5^ It is dosed at one to two mg/kg intravenous (IV) over three to five minutes. The time to peak concentration for IV methylene blue is 30 minutes, and the onset of action for the reduction of methemoglobin is 30–60 minutes.^6^ Clinical response should be observed within one hour.^8^ The dose may be repeated at one mg/kg if methemoglobinemia does not resolve within 30 minutes to one hour, or if the patient’s clinical status does not improve.^6^

At low concentrations, methylene blue enhances the reduction of methemoglobin to hemoglobin. It is converted to leukomethylene blue inside the red blood cell by erythrocyte methemoglobin reductase in the presence of nicotinamide adenine dinucleotide phosphate (NADPH). Leukomethylene blue is a reducing agent, and both compounds contribute to the reduction of methemoglobin in red blood cells.^5^ Methylene blue serves as a cofactor to accelerate NADPH-MetHemoglobin reductase. The ferric ion (Fe^3+^) on methemoglobin is converted back to its oxygen-carrying state (Fe^2+^), resulting in hemoglobin (see Figure). 7

An alternative pharmacological option is high-dose ascorbic acid, colloquially known as Vitamin C. The mechanism of ascorbic acid in methemoglobinemia is purported to be due to an antioxidant effect, which is directly proportional to plasma concentrations. High doses (up to 10 grams) are required to rapidly achieve the necessary levels to achieve methemoglobin reduction. One notable concern for this treatment is that high doses increase urinary excretion of oxalate, which has been reported to cause oxalate nephropathy in patients with renal impairment.^9^

In a case series reported from an institution where methylene blue was not available, five pediatric patients were successfully treated for methemoglobinemia with ascorbic acid. All patients required three or four doses of 1.5 to two grams intravenously, and their methemoglobin levels were reduced to non-toxic levels within 24 hours of treatment.^10^

No randomized controlled trials have been published to directly compare the two treatments, but methylene blue has traditionally been the agent of choice because of its rapid onset. Methylene blue has been shown to reduce methemoglobin to non-toxic levels within 10 to 60 minutes while ascorbic acid may require 24 hours to achieve the same effect. Additionally, ascorbic acid requires multiple doses much more frequently.^9^,^10^ Finally, in severe cases of methemoglobinemia, exchange transfusion and hyperbaric oxygen can also be used as adjunctive or monotherapy.^11^,^12^

## CONCLUSION

This case demonstrates the importance of considering methemoglobinemia in any patient with toxic ingestion of unknown contents, as well as those whose saturations do not improve with supplemental oxygen. It also demonstrates how precipitously patients with methemoglobinemia can desaturate over a short period of time. Methemoglobin should be considered in toxic ingestions, especially in substances that are not typically ingested with unknown contents. Lastly, if methemoglobinemia does present and if methylene blue is given, patients tend to respond to therapy within the hour and oxygen saturations can be expected to improve rapidly.

Documented patient informed consent and/or Institutional Review Board approval has been obtained and filed for publication of this case report.

## Figures and Tables

**Figure:**
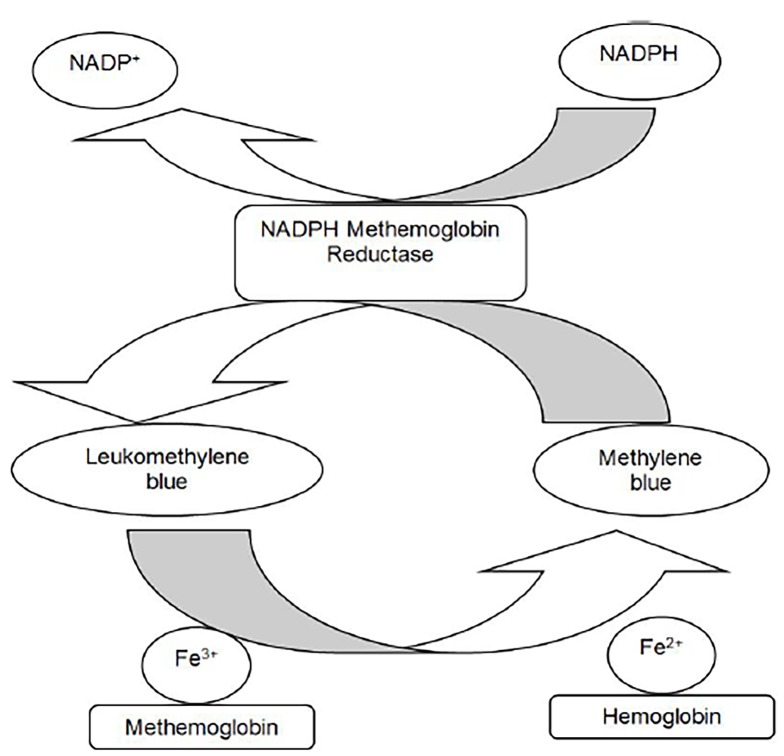
Methemoglobin process of metabolism. *Fe^2+^*, ferrous state; *Fe^3+^*, ferric state; *NADP^+^*, nicotinamide adenine dinucleotide phosphate; *NADPH*, reduced state of nicotinamide adenine dinucleotide phosphate.
